# Analysis and Algorithmic Generation of Hepatic Vascular Systems

**DOI:** 10.1155/2012/357687

**Published:** 2012-09-26

**Authors:** Lars Ole Schwen, Tobias Preusser

**Affiliations:** ^1^Fraunhofer Institute for Medical Image Computing MEVIS, Universitätsallee 29, 28359 Bremen, Germany; ^2^School of Engineering and Science, Jacobs University, Campus Ring 1, 28759 Bremen, Germany

## Abstract

A proper geometric model of the vascular systems in the liver is crucial for modeling blood flow, the connection between the organ and the rest of the organism. In vivo imaging does not provide sufficient details, so an algorithmic concept for extending measured vascular tree data is needed such that geometrically realistic structures can be generated. We develop a quantification of similarity in terms of different geometric features. This involves topological Strahler ordering of the vascular trees, statistical testing, and averaging. Invariant features are identified in human clinical in vivo CT scans. Results of the existing “Constrained Constructive Optimization” algorithm are compared to real vascular tree data. To improve bifurcation angles in the algorithmic results, we implement a postprocessing step calibrated to the measured features. This framework is finally applied to generate realistic additional details in a patient-specific hepatic vascular tree data set.

## 1. Introduction

The most important link between the liver and the rest of the organism is the blood flow through three vascular systems [1, 2]. These include two supplying systems, the portal vein (PV) providing venous blood drained from the digestive system and hepatic artery (HA) providing arterial blood, as well as one draining system, the hepatic vein (HV). The bile duct is a fourth vascular system which transports the secreted bile from the bile canaliculi out of the liver into the cystic and common bile duct finally leading into the duodenum.

On the one hand, for the functioning of the liver metabolism a proper blood supply and drainage are essential. In fact, the liver receives about 25% of the cardiac blood output, which amounts to about 100 mL/min per 100 g net liver weight [3]. On the other hand a variety of pathological conditions result in impaired blood flow conditions. For example cirrhosis decreases total hepatic perfusion while increasing the fraction of arterial blood [4]; metastases also lead to an increased arterial fraction [5]. Also, it is known that the blood flow plays a major role in the regeneration capabilities of the liver. In summary the blood flow plays a central role in the liver function and understanding blood flow, and its regulation can be seen as a key to understanding liver physiology and pathology.

Biophysical modeling and simulation have become powerful tools in analyzing and understanding the behavior of complex dynamical systems or to predict future states of such systems without actually performing the corresponding experiments. Such modeling and simulation of physiological processes in the human body do not only have impact on the basic science of understanding life but also direct consequences ranging from pharmaceutical developments up to improved quality in surgical interventions.

In order to properly model and simulate the metabolic function of the liver, it is crucial to have an appropriate model of the blood transportation systems. In a multiscale model [6], the range between the whole organ (e.g., pharmacokinetic models such as [7, 8], and the individual lobules [9–11] is covered by the vascular systems).

For modeling and understanding physiological processes, the level of detail needed in the vascular structures depends on the spatial heterogeneity and the scale of the process being considered. For planning liver resection surgery (see, e.g., [12]), one main task is to determine the territories supplied by large vessels. For this purpose, details available from in vivo imaging are sufficient. Certain liver diseases were observed to be spatially inhomogeneous, for example, chronic hepatitis and cirrhosis [13], fibrosis [14, 15], and steatosis [16]. In this case, a multiscale model considers representative volume cells, consisting of groups of lobules, of the liver sufficiently small such that their properties can be assumed to be more or less homogeneous. The vascular structures then need to be sufficiently detailed to properly reflect supply and drainage of such representative volumes. Lobules, their internal sinusoidal network and a potential zonation of hepatocytes, can and should be viewed on a separate scale in a multiscale simulation framework.

However, current imaging and image processing techniques are not capable of resolving the full vascular system in human livers. Thus, studying the vascular structures from coarse to fine scale, from the portal vein to the finest hepatic units, the sinusoids, is not directly possible.

In vivo CT scans of the human liver provide a resolution of a few hundred micrometers. Ex vivo corrosion casting is an established technique requiring huge skills. If not prepared in situ, however, a deformation of the organ needs to be accepted. Corrosion casts are obviously smaller than whole bodies and permit higher doses of radiation and thus provide better image data, but only few of them are available.

Microscopy generates optical images at much higher resolution which are capable of resolving intralobular sinusoids, but the images are essentially only 2D. Reflectance confocal microscopy [17] or fluorescence confocal microscopy allows optical imaging a few 100 *μ*m below the surface without physical sections. Histological serial slices can be created and scanned optically, but the cutting process introduces physical artifacts that can only be remedied to a limited extent by image processing (registration, inpainting).

To bridge this resolutional gap, algorithms for generating vascular systems can be applied, thus allowing to build full three-dimensional vascular trees that permit modeling and simulation of liver blood flow from the coarsest to the finest scales. However, to assess and improve the quality of such algorithmically generated structures, that is, to determine whether they are sufficiently realistic for modeling purposes, requires better techniques than merely a visual comparison.

The purpose of this paper is twofold. On the one hand, we describe a method to quantify the difference between structural and geometric features of different vascular structures in Section 3. We will compare a significant number of real contrast-enhanced liver CT data sets and several corrosion casts. Thus we will be able to quantify the similarity of hepatic vascular structures over a large group of individuals. On the other hand, we use these findings to improve an existing algorithmic method to more closely resemble hepatic vascular structures found in humans in Section 4. After this calibration, the method is finally applied to extending measured vascular networks by more details than we can obtain from the image data. While we restrict the description and analysis to one of the vascular systems of the liver in this work, the framework is generic and can be applied to other organs as well. Moreover, the extension to more than a single vascular structure is possible and currently being investigated.

## 2. Review of Related Work

Vascular structures have been studied for a variety of human organs such as lungs [18, 19], hearts [20, 21], retinas [22], and livers [23] as well as in whole rats [24] and animal organs such as cat lungs [25], pig hearts [26–28], rat hearts [29], and rat kidneys [30]. Even though this section provides a relatively detailed overview on existing work, it is by no means meant to be an exhaustive literature review.

Borrowing vocabulary from graph theory (see, e.g., [31]), many vascular systems can be described as trees consisting of nodes and edges. In the tree hierarchy we will use the terms “parent” and “daughter” edge and permit only strictly bifurcative trees; that is, each edge has exactly zero or two successors, with a single edge incident to the root node. In most cases we are not merely interested in the topology but also the geometry of vascular structures, so nodes are typically assigned a geometric position and edges a radius. As the edges play a more important role in our considerations than bifurcations/nodes, we assign geometric quantities to edges in our analysis. To avoid confusion with “Couinaud segments” going back to [32], we avoid the term “segment” when dealing with vascular networks.

**Topological Classification**

The simplest topological classification for edges in a tree is the generation number [33] (also named “bifurcation level” [20], “level” [24], “Weibel order” [34]) which, however, is incapable of distinguishing the “importance” of edges in terms of their supplied subtree). The most commonly used classification is the “Strahler” order (e.g., in [19, 35, 36] and many more articles) originally used to classify rivers [37, 38], a measure nondecreasing from leaves to the root. More precisely, edges leading to leaves are assigned order 1; parent edges are assigned the larger order of its daughters (if they differ) or the two daughters' order plus 1; see also Figure 2. While it may seem problematic at first glance to start at the smallest edges with highest measurement uncertainty, this scheme is actually very robust. Compared to the more intuitive generation counting, Strahler ordering is a “major improvement […] because it takes into account the asymmetric branching pattern” [39]. Two edges near the root have the same generation number even if one is “small” and leads to a leaf node and its sibling supplies a “large” subtree; see also [25].

The Strahler ordering scheme has been refined by also including radius information (e.g., in [34, 39, 40]). The order at a bifurcation is then only changed if there is a sufficient increase of radius. In our analysis we chose not to use this type of levels because it is no longer strictly topological and because the radius data in our data sets is not very accurate [41].

**Geometrical Features**

The two most obvious geometrical features for tubular edges are their radius and length [42–44]. At bifurcations, different angles can be considered: the angle between the continued parent edge and either of the daughters [28, 45, 46], the angle between the two daughters [29], the inclination of one of the edges with respect to the plane spanned by the other two (cf. [43]). In [47], Voronoï cells of the leaf nodes are considered, and their asphericity is measured. They can be combined to supplied volumes of edges, but in any case the resulting general polyhedra are not easily comparable.

From the previous “natively” geometric features, derived geometric features can be computed. From the radius, we can compute the radius ratio to the parent [28], the radius asymmetry between larger and smaller daughter radius (“branching ratio” in [20], “bifurcation index” in [28, 48]) or vice versa [42]; cross-section areas and analogous cross-section area ratios [20, 48] and asymmetries [49]. Moreover, a “bifurcation exponent” *γ* can be computed for *r*_p_^*γ*^ = *r*_1_^*γ*^ + *r*_2_^*γ*^ where *r*_p_ is the parent radius and *r*_{1,2}_ are the daughter radii; see, for example, [48, 50]. The exponent *γ* = 3 is an optimal trade-off between power dissipation for moving the blood and metabolic cost for maintaining the blood vessel wall if laminar Poiseuille flow can be assumed [51, 52]. While this is a property of a single vascular edge, flow (mass) conservation at bifurcations allows considering *γ* to be a property of a bifurcation. An alternative value *γ* = 2.7 is used in [53] as it minimizes vascular wall material [54] and was measured in [55]. The value *γ* = 2.55 minimizes the reflection of pulse waves at bifurcations [56].

Based on the purely geometric features, derived features have been investigated, computed according to additional assumptions. These include pressure drops, inertance, compliance [57], perfusion heterogeneity and asymmetry of supplied volume [58], edge flow, exit pressure and transit times [59], additional compliances [60], pressure profiles [61] and perfusion inhomogeneity in surrounding tissue [61, 62], and wall shear, internal pressure, and circumferential tension [63]. Theoretically optimal angles are discussed in [64, 65].

Various geometric features were determined depending on the bifurcation order/generation number [20, 29, 44] or on the Strahler order [18, 19, 30, 66] and on radius-based Strahler orders [18, 34, 58]. Also, the dependency of various features on edge radii, which are a noninteger quantity was investigated [33, 59, 67–69]. Also, relations between different features were discussed in [21, 44, 50], to name just a few. Software for analyzing vascular trees is presented in [29]. Self-similarity and other fractal properties were frequently observed and investigated; see, for example, [70–75].

Our geometric analysis will be based on the strictly topological Strahler order scheme and only take into account purely geometric features so that there is no dependency on additional physical or physiological assumptions.

**Constructive Optimization**

Different types of algorithms for generating vascular structures have been presented in the literature. One class of algorithms is based on constructive optimization, that is, determining vascular networks satisfying physiological optimality criteria (e.g., minimal intravascular volume) for a given supply requirement. This requirement is typically given by a set of pseudorandomly distributed leaf nodes interpreted as connections to lobules on the next finest geometric scale. The most prominent approach here is “Constrained Constructive Optimization” (CCO) [61, 69, 76, 77]. These were originally developed for supplying a convex domain (organ); an extension to the nonconvex case is presented in [78]. In contrast, “Global Constructive Optimization” [79, 80] performs a multiscale optimization finding an optimal tree for all leaf nodes at the same time.

The basic principle of CCO is to construct a strictly binary tree by adding one leaf node at a time to an initial tree, each time introducing an optimal bifurcation. Thus, CCO can be seen to be driven by the assumption of equal in- or outflow at all leaf nodes representing constant supply/drainage for each lobulus. Moreover, at bifurcations the radii are balanced such that the flow resistance according to the Hagen-Poiseuille law [81] is equal for both subtrees. This finally results in equal exit pressures at the leaf nodes.

**Deterministic Geometric Construction**

Other authors use a deterministic geometric construction using Cartesian [82] or hexagonal [53] cell-based models or constructions inspired by self-similar (fractal) or area/space filling objects [43, 83–86]. Such approaches benefit (in terms of algorithmic complexity) from avoiding optimization problems. The resulting vascular networks, however, suffer from an artificial overall structure, which is even visually perceptible, so that they are not appropriate for general models.

**Angiogenesis-Based Construction**

**Figure 1:**
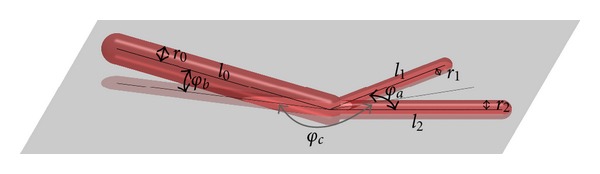
A bifurcation with the radii, lengths, and angles considered for our geometric analysis.

Yet another approach is modeling angiogenesis, the actual process by which vascular structures grow. This type of approach requires more involved models and algorithms than basic optimality conditions. Earlier results in this area [46, 53, 82, 87] exhibit a visually artificial structure or “somewhat stylized appearance” [82, Figure 1]. More recent work includes [88, 89], the latter combining introducing new vascular edges due to angiogenesis with subsequent geometric optimization of the vascular tree. Grid-based methods typically produce visual geometric artifacts reflecting the grid used. To us there seems no easy way of introducing parameters in these algorithms for being able to calibrate them to better match geometric features measured in the vasculature of human livers. One could combine angiogenesis models with geometric parameters by changing the way how new vascular edges form. Instead of only considering gradients of angiogenetic factors generated by ischemic cells, also properties of the existing vascular edge for which a new bifurcation is to be introduced, on the branching angles of that bifurcation or on other properties of the existing vascular structure, could be taken into account. This would, however, involve additional assumptions for the model and parameters in the algorithmic implementation which are not easily observed experimentally.

In this paper we base the construction of vascular structures on CCO as this is an established technique and avoids the drawbacks of deterministic or angiogenesis-based methods.

## 3. Morphometry of Hepatic Vascular Systems

In this section, we aim at quantifying the similarity between different vascular networks. We first describe the specimens used for this study. A similarity measure is introduced and results are presented.

### 3.1. Data Acquisition

For the geometric analysis, two types of data were used. Corrosion casts of 6 human PVs from [86, 90] and described therein plus one additional PV data set were used. Only 3 corrosion cast HV data sets were available from these studies, not enough for a robust statistical analysis, so we ignored them for our purposes. CT scan image data was segmented and skeletonized by a semiautomatic procedure described in [23]. This yields a graph representation of the vascular structures with curved edges of nonconstant radius. Moreover, 167 PV and 165 HV vascular trees were obtained from clinical in vivo CT scans using contrast agent, for which image preprocessing the same procedure was applied. Edges of the HA scans are thinner, and the extracted data is less reliable than for the other structures, so they were excluded from our consideration. As the HA is essentially parallel to the PV (and bile ducts) and is responsible for roughly one-third of the PV flow (albeit of different composition), a separate analysis of these was considered of minor importance. The bile duct is not considered here.

Depending on the quality of the image data and the preprocessing steps the graph representations were further modified because different properties of the tree may violate the strictly bifurcative property needed for our analysis. Isolated nodes, monofurcations, or multifurcations (edges with more than two daughters) may be present. Loops may exist in two different flavors, either as two distinct paths between two nodes in edge-parent direction (improper loops) or as nontrivial paths in edge-parent direction from a node to itself (proper loops). Improper loops contain at least one node with two incident parent edges. The root node may have more than one incident daughter edge. Thus a correction step is needed to make the extracted graphs suitable for our further analysis.

In this correction step, existing trivial edges (initial node equals terminal node) are removed. Nodes are then tested for the number of incident parent edges. If multiple parent edges coming from the same initial node are found, their radii are combined such that the cross-section area is preserved. For multiple parent edges with different initial node, one with maximum radius is preserved, and the others are removed. Next, monofurcations are removed and multifurcations are split by again introducing trivial edges. Removing monofurcations requires averaging the radii of the two edges involved. As these radii are already estimated radii from the image data, this is only a slight modification comparable to inaccuracies introduced by the skeletonization. Introducing trivial edges does not affect the geometry of the vascular structures at all.

Next, we test for the existence of proper loops which, however, did not occur in our data sets. Any isolated nodes are removed and we finally copy the subtrees for all root nodes found, introducing trivial edges if necessary such that root nodes only have one incident daughter edge.

When dealing with corrosion casts, we must note that deformations of the corrosion casts not produced in situ particularly affect bifurcation angles for the larger edges. Also we must be aware of the fact that radius information is not very accurate in the graphs [41] and resulting trees. This limitation is mainly due to the fact that the vascular structures are only a few voxels wide in the image data. Thinner vascular segments—if they are present in the corrosion cast or filled by contrast agent at all—are not visible in the image data. The algorithms used in the semiautomatic image processing have a high hardware demand and are currently limited to processing images of about 70 megavoxels. Simpler, fully automatic image processing techniques do not produce satisfying results because the connectivity of the individual networks, but separateness of separate networks, if present in one image data set, cannot be obtained reliably.

### 3.2. Topological and Geometrical Quantification

**Figure 2:**
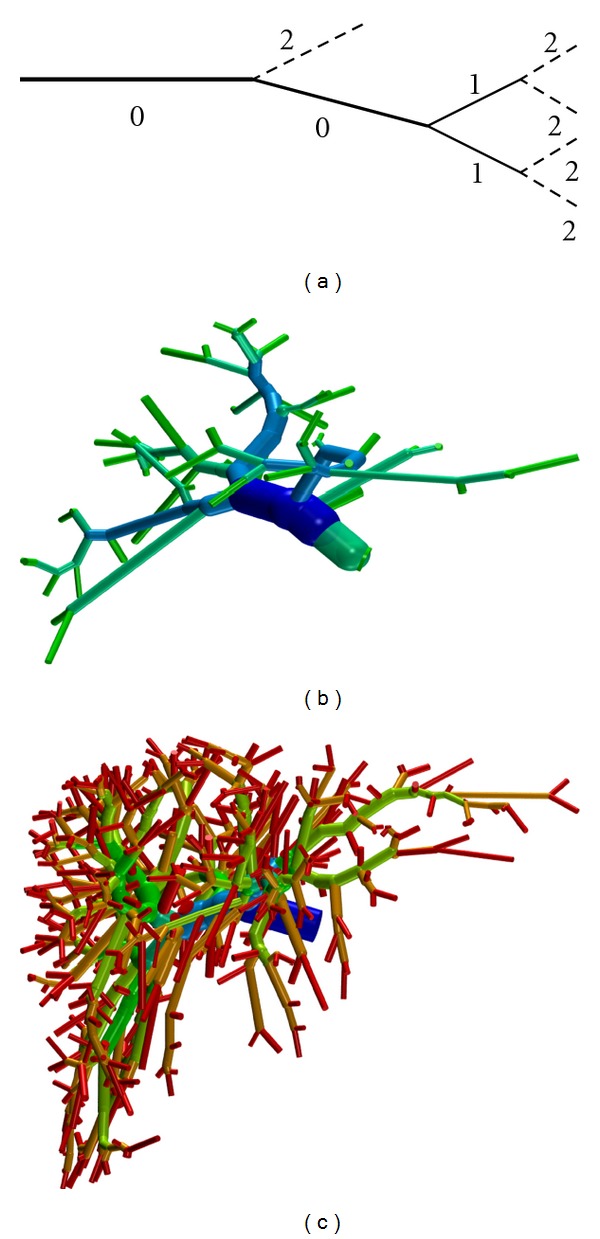
The (a) sketch shows the definition of Strahler* orders: the order decreases towards the root if two edges of the same order meet, else the minimum is used, and the values are shifted such that the root edge has order 0. Images (b) and (c) show Strahler* orders (HSV color coded from blue = 0 to red = 5) for two of the clinical PV data sets.

 As we have different sources of image data (contrast enhanced CT, CT of corrosion casts) with different image quality, we will be comparing vascular structures with potentially different level of detail resolved. Thus, Strahler orders of the respective root edges differ, and edges of a given Strahler order play a different role in different trees. We hence introduce a the notion of Strahler*order, assigning to each edge the difference between the Strahler orders of the root and the current edge; see Figure 2.

For the analysis of the vascular structures we will focus on the following geometric features for each edge in the tree. For an overview of the many possible geometric properties and of what has been considered in the literature we refer to Section 2.

**Radii**

Each edge has a radius *r*, a ratio *η*_*r*_ = parent radius /current radius, except for the root edge. Moreover, for each nonterminal edge there is a ratio *σ*_*r*_ = max⁡ daughter radius/min⁡ daughter radius. Moreover, the bifurcation exponent *γ* described previously is considered a property of the parent edge.

**Lengths**

Each edge has a length *l*, *η*_*l*_ and *σ*_*l*_ are defined as the obvious analogues of *η*_*r*_ and *σ*_*r*_.

**Angles**

A bifurcation is fully described by three angles. Without loss of generality, assume the two daughter segments lie in the *xy* plane such that their angle bisector is aligned with the *x* axis. Then let *φ*_*a*_ be the angle between the two daughter edges, let *φ*_*c*_ be the angle between the projection of the parent edge to the *xy* plane and the *x* axis, and let *φ*_*b*_ be the inclination of the parent edge to the *xy* plane; see Figure 1. Note that these angles can be computed as properties of the parent without distinguishing the two daughters.

If, at a given bifurcation, a geometric feature cannot be computed, the value is not considered for the evaluation later on. Hence, the number of bifurcations actually considered for different features may vary.

Fractal properties, despite being interesting for individual, were not considered for a comparison later on. Furthermore, features computed from the purely geometric features were not considered such as to avoid redundancy and/or dependency on physical or physiological assumptions. These could be interesting in later studies.

### 3.3. Quantification of Similarity

 In order to obtain a scalar measure of similarity within one set *𝒯*_m_ of vascular trees (e.g., measured trees) or between two sets *𝒯*_m_ and *𝒯*_g_ of vascular trees (e.g., measured versus generated ones), we perform six steps in which we successively compute

1. Strahler* orders for each segment of each tree and the applicable geometric features,
2. histograms of the geometric features described previously per Strahler* order for each tree,
3. a binary decision of similarity for each geometric feature and each Strahler* order between each pair of trees (within either *𝒯*_m_ × *𝒯*_m_∖Δ or  *𝒯*_m_ × *𝒯*_g_);  we are then interested in the ratio (number of pairs classified similar) over (number of all pairs), still for each geometric feature and each Strahler* order,
4. a weighted average of these ratios over the Strahler* orders for each geometric feature,
5. weighted averages for the radius, length, and angle features;
6. a weighted overall average.

**Statistical Interpretation**

The computation in Step 1 is straightforward and described in Section 3.2. For the remaining steps, we consider the values as samples of an unknown underlying probability distribution, for which we compute the (empirical) cumulative distribution function (CDF) in Step 2. Note that we do not use probability densities because the CDFs are directly used in subsequent statistical testing and because they avoid binning when plotting the data.

**Testing for Similarity**

For two CDFs corresponding to the same geometric features of edges of fixed Strahler* order of two different vascular trees we use the two-sample Kolmogorov-Smirnov test [91, 92] (KS test), to check the null hypothesis that the two CDFs have the same underlying distribution. In Step 3 we classify two trees *T*_0_, *T*_1_ as “similar” (in terms of this feature and Strahler* order, written as *T*_0_ ~ *T*_1_) unless the test rejects the null hypothesis at significance *P* = 0.05. Note that there is no particular reason for choosing this value here; other than that is is very common [93]. In comparison to other statistical tests, for example, the Cramér-von Mises test [91], the KS test has the advantage of being independent of scaling in the values of the underlying distributions.

Let us first consider the case of one set *𝒯* of vascular trees, where we are interested in the similarity among, for example, the clinical PVs. This will also be referred to as the *single-population case*. A similarity ratio for a fixed geometric feature *f* and Strahler* order *s* is then computed as

(1) $$\begin{array}{c} \;\;m_{f,s}\left(𝒯\right):=\frac{\#\left\{\left(T_{i},T_{j}\right)\in 𝒯\times 𝒯\;|\;T_{i}\sim T_{j},\;T_{i}\neq T_{j}\right\}}{\#\left\{\left(T_{i},T_{j}\right)\in 𝒯\times 𝒯\;|\;T_{i}\neq T_{j}\right\}}, \end{array}$$

Where ~ denotes classification as “similar” in terms of *f* and *s* in the sense defined above.

If we are interested in the similarity between, for example, a set *𝒯*_m_ of measured vascular trees and a set *𝒯*_g_ of generated trees, we define a similarity ratio as

(2) $$\begin{array}{c} \;\;m_{f,s}\left({𝒯}_{\text{m}}{𝒯}_{\text{g}}\right):=\frac{\#\left\{\left(T_{0},T_{1}\right)\in {𝒯}_{\text{m}}\times {𝒯}_{\text{g}}\;\;|\;T_{0}\sim T_{1}\right\}}{\#\left\{\left(T_{0},T_{1}\right)\in {𝒯}_{\text{m}}\times {𝒯}_{\text{g}}\right\}} \end{array}$$

and refer to this as the *two-population case*.

Note that due to the nature of statistical testing, we cannot expect these similarity values to be larger than 1 − *P*. This also holds for the weighted averages in the following.

Averaging over Strahler* Orders

In order to average over the Strahler orders in Step 4, but still considering a fixed geometric feature *f*, we wish to take into account the number of edges present in the tree at different Strahler* orders. Since different vascular trees in the population considered may have different level of detail, we restrict the averaging to those Strahler* orders ($s<\tilde{s}$) below which at least half of the trees actually have edges. The weighted average ${\overset{-}{m}}_{f}(𝒯)$ is obtained as

(3) $$\begin{array}{c} n_{s}\left(𝒯\right):=\#\left\{T_{i}\in 𝒯\;|\;T_{i}∋\text{edges}\;\text{of}\;\text{order}\;s\right\}, \end{array}$$

(4) $$\begin{array}{c} {\overset{-}{m}}_{f}\left(𝒯\right):=\frac{\sum_{s<\tilde{s}}^{}w_{s}\left(𝒯\right)\cdot m_{f,s}\left(𝒯\right)}{\sum_{s<\tilde{s}}^{}w_{s}\left(𝒯\right)}. \end{array}$$

In the two-population case, the maximal Strahler* order $\tilde{s}$ is the maximal order at which at least half of the trees in both populations actually have edges and the averaging weights are the geometric means of the same values as before. The weighted average ${\overset{-}{m}}_{f}({𝒯}_{\text{m}}{𝒯}_{\text{g}})$ for a fixed geometric feature *f* is obtained as

(5) $$\begin{array}{c} \tilde{s}\left({𝒯}_{\text{m}},{𝒯}_{\text{g}}\right):=\min\left(\tilde{s}\left({𝒯}_{\text{m}}\right),\tilde{s}\left({𝒯}_{\text{g}}\right)\right), \end{array}$$

(6) $$\begin{array}{c} {\overset{-}{m}}_{f}\left({𝒯}_{\text{m}},{𝒯}_{\text{g}}\right):=\frac{\sum_{s<\tilde{s}}^{}w_{s}\left({𝒯}_{\text{m}},{𝒯}_{\text{g}}\right)\cdot m_{f,s}\left({𝒯}_{\text{m}},{𝒯}_{\text{g}}\right)}{\sum_{s<\tilde{s}}^{}w_{s}\left({𝒯}_{\text{m}},{𝒯}_{\text{g}}\right)}. \end{array}$$

**Averaging over Features**

For averaging over features in Step 5 in the one-population case, we use the arithmetic mean to quantify an average similarity. In the two-population case, we use the values ${\overset{-}{m}}_{f}({𝒯}_{\text{m}})$ as averaging weights so that invariant features in the measured trees are weighted stronger than noninvariant ones, such that we can quantify “similarity where it is expected.” The two numbers should hence not be compared directly. Averages ${\overset{-}{m}}_{F}$ over a set *F* of features are defined as

(7) $$\begin{array}{c} {\overset{-}{m}}_{F}\left(𝒯\right):=\frac{1}{\#F}\sum_{f\in F}{\overset{-}{m}}_{f}\left(𝒯\right), \end{array}$$

(8) $$\begin{array}{c} {\overset{-}{m}}_{F}\left({𝒯}_{\text{m}},{𝒯}_{\text{g}}\right):=\frac{\sum_{f\in F}^{}{\overset{-}{m}}_{f}\left({𝒯}_{\text{m}}\right)\cdot {\overset{-}{m}}_{f}\left({𝒯}_{\text{m}},{𝒯}_{\text{g}}\right)}{\sum_{f\in F}^{}{\overset{-}{m}}_{f}\left({𝒯}_{\text{m}}\right)} \end{array}$$

in the one- and two-population cases, respectively.

Finally, in Step 6 we average over different sets *F* of features (related to radii, lengths, and angles, resp.) in such a way that the different numbers #*F* of features per set are taken into account. In the one- and two-population cases we average as follows:

(9) $$\begin{array}{c} w_{F}:=\frac{\sum_{f\in F}^{}{\overset{-}{m}}_{f}\left(𝒯\right)}{\#F}, \end{array}$$

(10) $$\begin{array}{c} \overset{-}{m}\left({𝒯}_{\text{m}},{𝒯}_{\text{g}}\right):=\frac{\sum_{F}^{}w_{F}\cdot {\overset{-}{m}}_{F}\left({𝒯}_{\text{m}},{𝒯}_{\text{g}}\right)}{\sum_{F}^{}w_{F}}. \end{array}$$

**Implementation of the KS Test**

The two-sample two-sided KS test [91, 92] involves three steps, computing the maximal difference between the two CDFs, rescaling it by the factor $\sqrt{n_{0}n_{1}/n_{0}+n_{1}}$ depending on the two sample sizes and evaluating the KS distribution to check whether it is smaller than the significance level. Evaluating the KS distribution requires a brief discussion.

Tabulated vales [94, 95] (e.g., [96] based on Monte Carlo simulations) for fixed significance levels were used traditionally, which lacks the possibility to use arbitrary significance levels if this is desired. The limiting KS distribution [97, 98] for large sample sizes

(11) PK(x)=1−2∑i=1∞(−1)i−1e−2i2x2=2πx∑i=1∞exp⁡−(2i−1)2π28x2

is the same in the one- and two-sample cases [99] with appropriate scaling factors for the sample size.

Evaluation of the distribution in the one-sample case for small samples is discussed in detail in [98]. A review of current software capable of doing two-sample KS tests reveals that *octave* [100, 101] and *root* [102, 103] use the limiting KS distribution regardless of sample size. For small samples, *R* [104] uses [105] for the one-sample test and an undocumented method for the two-sample test, which seems to be a generalization of [106] to samples of different cardinality.

We performed own Monte Carlo Simulations creating many small samples with (uniformly distributed or the sum of two uniformly distributed) random numbers, obtained using the Mersenne Twister [107], and computing the KS values for them. These should be uniformly distributed in [0,1], regardless of the distribution of the sample if all samples are drawn from the same distribution. For the limiting distribution, we confirmed the known “overestimating effect” [108]. Using the one-sample implementation of [98], available as a supplement to that paper, in the two-sample case showed only a slight overestimation of the distribution. The code used in *R*, however, produced accurate results. The numerical computation of the CDFs and their differences should be performed with at least IEEE double accuracy.

### 3.4. Results

Results of the topological analysis are shown in Tables 1 and 2 show that how many of the vascular trees considered had edges at different Strahler* orders and how many edges there were on average, as well as a histogram of the connectivity between different Strahler* orders. The maximal Strahler* order in the clinical CT scans and the corrosion cast data sets is 6 even though the corrosion casts contain more edges, in particular of high Strahler* order, which is also reflected in the connectivity histogram. For a strictly binary tree with 2^s*^ leaf nodes, these histograms would show only connections between subsequent levels, approximately 0.5^s*−*s*^ · 100% for the connection between levels *s* − 1 and *s*.

**Figure 3:**
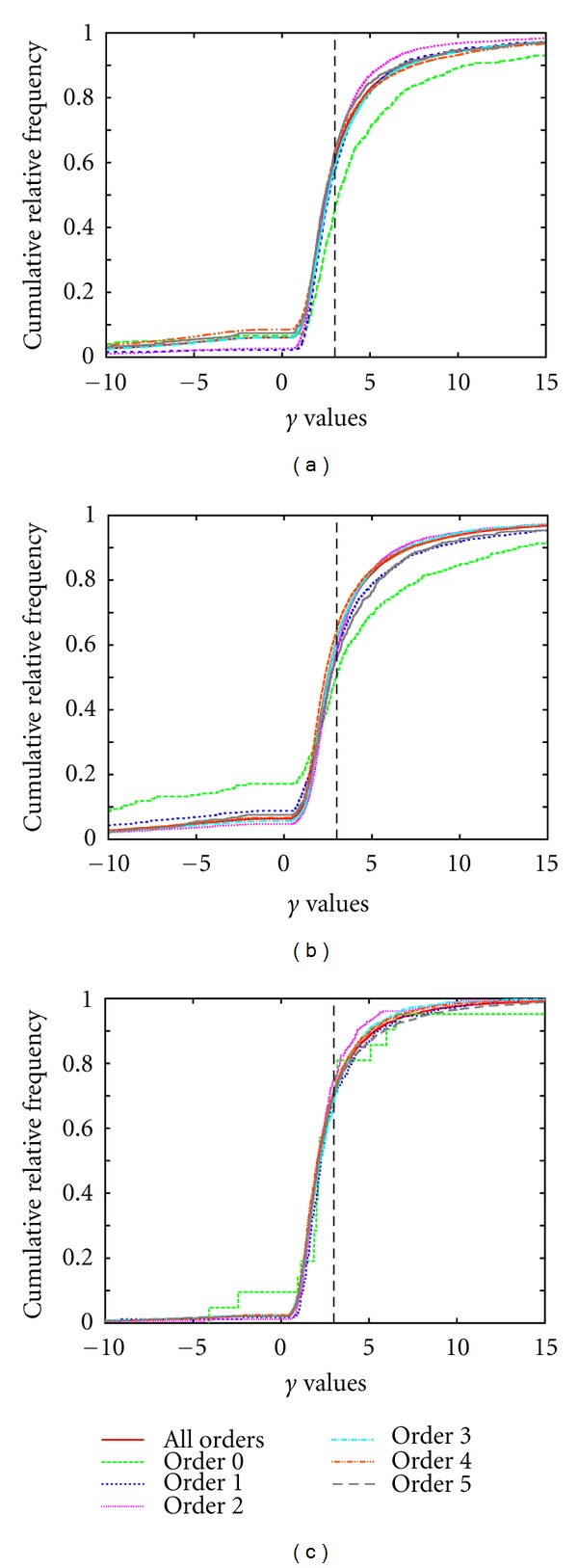
Overall histograms of the bifurcation exponents *γ* for our clinical PV (a), HV(b), and corrosion cast PV (c) data sets for different Strahler* orders.

The cumulative distributions of the bifurcation exponents *γ* for all the data sets considered are shown in Figure 3. These turn out to be very similar for all Strahler* orders >0 but differ between the clinical PV and HV as well as between clinical and corrosion cast PV data sets. Radius data, in particular for thin radii at high Strahler* orders, is not very accurate, so part of these distributions may be due to imaging and image processing inaccuracies. However, larger radii are more robust and exhibit the same distribution.

The results of the similarity analysis for our data sets are listed in Table 3. We can observe that the similarity among HV trees is slightly smaller than the similarity among PV. The similarity among the corrosion cast PV data sets is much smaller; this is probably due to the small number of specimens considered and the different conditions under which they were manufactured. Comparing clinical and corrosion cast PV, radii and absolute lengths are relatively different whereas angles are only slightly less similar than among the clinical PV. Moreover, Table 3 shows that the similarity between PV and HV is comparable to the similarity within each of the two populations, except for the angle features.

The averaging procedure over Strahler* orders for two fixed geometric features in the one- and two-population cases is shown in Table 4. The plots of the CDFs also illustrate that not only the average difference but also number of samples (visible as vertical steps in the graph) influences the similarity percentages *m*_edge radii,*s*_(*𝒯*) and *m*_*γ*,*s*_(*𝒯*_0_, *𝒯*_1_)—which is what the statistical test is meant to take into account. The decrease of the average radius with increasing Strahler* order, see, for example, [86], and other dependencies of geometric quantities on the topological orders can be confirmed in the detailed results but were not within the scope of the statistical comparison in this study.

## 4. Algorithmic Generation of Vascular Structures

In this section, we first discuss our implementation and application of standard CCO. We then propose improvements and quantify their effects finally showing applications of the improved scheme.

### 4.1. Constrained Constructive Optimization

 First, we consider CCO as described in the literature [61, 69, 76, 77]. Since livers are of nonconvex shape, we use a penalty approach [109, Section 4.2] inspired by [78]. We penalize the location of bifurcation nodes lying outside the organ domain by the squared distance from the organ scaled by a constant such that our objective function becomes

(12) $$\begin{array}{c} F\left(T\right)=\sum_{\text{edges}\;e}l\left(e\right)r^{\lambda }\left(e\right)+C\sum_{\text{nodes}\;n}\text{dis}{\text{t}}^{\text{2}}\left(n,\text{organ}\right) \end{array}$$

with *C* = 42 mm^*λ*−1^ if the unit of lengths is mm. The node-based penalty approach clearly only considers the two endpoints of a cylinder and ignores its radius, but we consider it appropriate as these cylinders are only meant to be an approximation of vascular edges which may be curved in reality. We choose *λ* = 2 but investigated different values of lambda (see also [110]).

The main task in CCO is to add one new leaf node to an existing vascular tree, finding an optimal new bifurcation. The topology of the optimal connection is not clear a priori; thus different new connections need to be tried and optimized geometrically. The geometric location of the newly introduced bifurcation is optimized using a gradient descent method with Armijo line search [109, 111]. The gradient is not evaluated analytically but using a centered difference quotient. This is because the radii of edges change, and not only locally so, as soon as a bifurcation is moved, as radii are rebalanced as described in Section 2. Leaving this out or only rebalancing radii after optimizing the position for fixed radii produces considerably different results.

It is known that a constant viscosity *μ* of blood can only be assumed for radii greater than 150 *μ*m due to the Fåhræus-Lindqvist effect [112]; see also [60] in the lung context. This is relevant because radii in a full vascular network range down to 10 *μ*m [50]. Changes in the effective viscosity can easily be integrated in the CCO procedure by evaluating the viscosity, which is needed when computing flow resistances for determining radii [63]; refer to [113] for a formula:

(13) $$\begin{array}{c} \mu \left(r\right)=\frac{\mu_{\infty }}{{\left(1+\delta /r\right)}^{2}} \end{array}$$

with *μ*_*∞*_ = 4.0 cP = 4 · 10^−3^ Pa s, *δ* = 4.29 *μ*m in the range *r* ∈ [4,150]  *μ*m. In order not to have a discontinuity in *μ*, we add a linear transition to *μ*_*∞*_ in the range 140 *μ*m ≤ *r* ≤ 160 *μ*m.

**Computational Workload**

To limit the computational workload for doing so, only a fixed number of nearby existing edges are tried (we heuristically determined that the 40 edges with closest midpoint to the new leaf node practically always contained the optimal new connection). Moreover, we first optimize with a rough stopping criterion (square root of the final tolerance), select the 20 best candidate topologies, then optimize these fully and select the optimum.

Without considering the Fåhræus-Lindqvist effect, the computational complexity for adding one new leaf node to an existing vascular tree with *n* nodes is *O*(*n*log⁡(*n*)), where factors log⁡(log⁡(*n*)) and higher logarithms are neglected here. This assumes that the number of gradient descent steps and Armijo steps in each gradient descent step is limited (we observed this in practice but cannot provide a rigorous proof for this claim) so that the number of radius rebalancing loops and cost function evaluations is bounded by a constant. The latter can be performed in *O*(*n*log⁡(*n*)) if appropriate elaborate caching of unchanged values is used. So the total workload for generating a vascular tree with *N* leaf nodes is *O*(*N* ^2^log⁡(*N*)). If, in addition, viscosity depends on radius due to the Fåhræus-Lindqvist effect, the radius rebalancing becomes more expensive because caching can no longer make use of relative radii of subtrees.

**Applying CCO in a Calibration Procedure**

Our goal is not to create vascular trees from scratch but to generate a higher level of detail in patient-specific given data sets so that in particular the coarse anatomic details are fixed and need not be generated algorithmically. To check how good generated vascular trees resemble measured ones, we thus proceed as follows.

**Figure 4:**
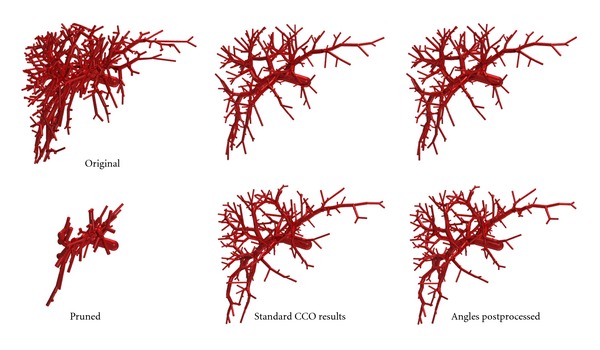
For the “dense” example in Figure 2, the (a, d) shows the preprocessed clinical PV tree before (a, b, c) and after (d, e, f) pruning to the coarse anatomic structures. Two resulting CCO trees before and after angle postprocessing are shown in the (b, e) and (c, f), respectively.

1. A given data set is loaded and preprocessed (made strictly bifurcative) as described in Section 3.1.
2. The vascular tree is pruned to containing only coarse anatomy by preserving the root and recursively its daughters as long as the initial node of the edge is outside the organ mask or the radius is larger than 0.25 times the maximum radius of the tree (see also Figure 4). The first condition makes sense because in the given data sets, the vascular structures start from outside the segmented organ mask.
3. Next, leaf nodes at pseudorandom positions (based on [107]) with a minimal distance between them are generated. Due to a pruning step later on, we here generate 1/0.27 times the actually desired number of leaf nodes (which in turn is chosen uniformly distributed in [150,350], thus being in a similar range as the data sets we wish to compare to).
4. One by one, these leaf nodes are added to the tree using CCO as described previously.
5. Finally, the leaf nodes of the “coarse anatomy” tree and additionally all generated leaf nodes are pruned from the tree, reducing the bifurcation level of the resulting data set. This is meant to reflect the fact that the clinical CT images were obtained at limited resolution, leaving out a lot of fine anatomy, and in particular not having leaf nodes near the boundary of the organ. The factor 1/0.27 above was determined heuristically to compensate for this pruning. Clearly more than one bifurcation level of nodes is missing in the clinical CT data sets (a human liver has 1 to 1.5 million lobules [114]), but in order to limit the computational workload for the calibration, we consider this one additional set of leaf nodes sufficient.
6. For each clinical CT data set, we generate 12 different CCO trees (different random seed leads to different sets of leaf nodes).

**Postprocessing for Geometric Analysis**

In order to avoid numerical artifacts due to very short segments in the subsequent geometric analysis and comparison to the measured vascular trees, very small edges shorter than a given threshold were contracted to length 0, partially resulting in Inf and NaN values being ignored in the analysis.

### 4.2. Results and Shortcomings of Standard CCO

In order to verify that *λ* = 2.0 minimizing intravascular volume in the CCO cost function indeed produces the best overall results, we apply the CCO procedure described previously for *λ* = 1.4,1.6,…, 2.6 to generate 7 different CCO trees for each of 16 of the clinical PV data sets. The effects of different *λ* on the appearance of the vascular trees generated by CCO were discussed in [47, 115]. As we can see from Table 5, *λ* = 2.0 produces better overall results than the smaller or larger values of *λ* considered here. Radii, for which we consider the measurements inaccurate, actually are least similar, but lengths are most similar for *λ* = 2.0. Angles are more similar for smaller *λ*, but these are postprocessed later on. Let us point out that the averages obtained for *λ* = 2.0 differ slightly from the values presented in Table 6 because only a subset of the clinical PV trees was considered here.

**Results of the Geometric Analysis**

Results of the comparison of generated and measured clinical PV and HV trees are shown in Table 6. From these data and the underlying cumulative histograms (not shown), a number of observations can be made.

Absolute radii are in a comparable but larger range than in the measured data sets, maybe indicating that the Strahler* ordering scheme is not the optimal topological classification. The radius decrease factor is bounded by 1 in the generated data sets which is not the case in the measured data. Radius asymmetry compares poorly, generated bifurcations are generally more asymmetric than the measured ones. The bifurcation exponent is fixed to *γ* = 3 in the algorithm, which is clearly an artifact compared to the measured distributions of *γ* (see Figure 3). However, the radius data from the measurements is not particularly accurate [41], so a high similarity between generated and measured radius-related features is not to be expected.

Absolute lengths are in a comparable but larger range than the measured data sets, similar to the absolute radii. Length decrease factors and length asymmetries are nicely similar between generated and measured trees.

Angles *φ*_*c*_ are nicely similar; *φ*_*a*_ in the generated data sets are significantly smaller than in the measured data. The same is true for the *φ*_*b*_, which is not surprising because the optimality criterion causes bifurcations to be flat. Note, however, that not all bifurcations are flat because CCO is not a global optimization procedure.

### 4.3. Improved CCO

As for further improving the standard CCO as described previously, we first tried to obtain a higher similarity of the bifurcation exponent *γ*, fixing which to 3 is clearly an artifact of the algorithm, as suggested by [20, 115]. This did not turn out to be useful, neither by fixing values according to the observed distribution (see Figure 3) already in the optimization procedure nor as a postprocessing step. In both cases, we obtained very unnaturally small absolute radii throughout the generated vascular trees. In case of postprocessing, the leaf edge radii are changed to a wide range, conflicting with the idea behind CCO that leaf nodes should provide homogeneous supply.

The poor similarity of the angles between daughters *φ*_*a*_ (which are too small on average) is improved by shifting the bifurcation point in the direction of the angle bisector as a postprocessing step. A value of 9% of the mean of the daughter edges was chosen for this purpose to fit the mean *φ*_*a*_ values of the generated vascular trees to the measured data.

Moreover, the poor similarity of the inclination angles *φ*_*b*_ is improved in a second postprocessing step. For this purpose, we consider one triangle per bifurcation, its vertices being the initial node of the parent and the terminal nodes of the daughter edges. We then compute the distance of the bifurcation point from this triangle relative to the longest edge of the triangle, also giving us a measure of nonflatness of the bifurcation for which the distribution can be computed separately for the clinical PV and HV data. As postprocessing, we modify the bifurcations in the generated vascular trees by moving the bifurcation points in normal direction to the triangles described before according to the nonflatness distribution, unless the triangle is degenerate. Note that this should not be done on a per-Strahler* order basis because data for the ranges we later want to apply the algorithm to is not available.

**Calibration Results**

The similarity results of the improved CCO output are listed in Table 6 next to the standard CCO results. Radii are not affected by the postprocessing. In particular, we do not rebalance radii throughout the tree afterwards; this turns out to further decrease similarity. Similarity in terms of length features becomes slightly worse by the postprocessing. Angle features, however, become much more similar, even the third angle *φ*_*c*_ for which an improvement was not aimed at by our postprocessing procedure.

The visual macroscopic difference introduced by the postprocessing is marginal; see Figure 4. Also notice that postprocessing the output of an optimization procedure decreases the optimality of the results. This cannot be seen in the cost function; actually the volume decreases by 5.5% on average (PV and HV) due to shorter daughter segments at bifurcations, but at the same time the flow resistance increases. This indicates that future work should also include “functional” features rather than merely geometric ones.

As we could already observe for the standard CCO case, generated HV is less similar to measured ones than it is the case for PV.

**Application of Improved CCO**

**Figure 5:**
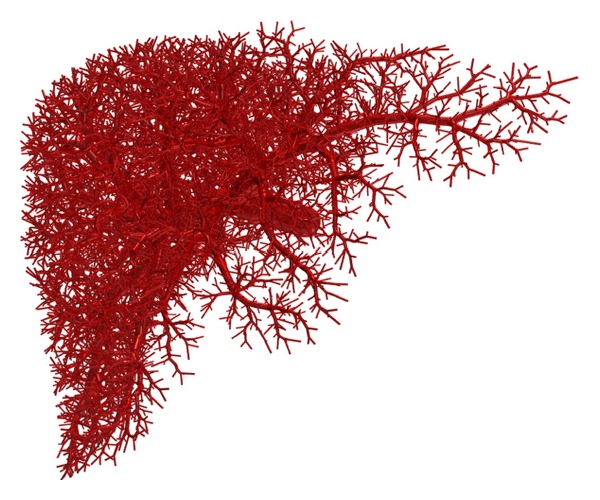
This vascular tree was generated applying the CCO algorithm including angle postprocessing to extend the clinical PV data set from Figure 4 without first pruning it. Each of the 10 000 leaf nodes in this setting is meant to supply about 125 lobules in the corresponding human liver.

Finally let us show an application of the improved construction algorithm. Starting from the dense PV example shown in Figure 2 (without pruning), we construct a tree supplying 10  000 leaf nodes. A human liver has about 1.0 to 1.5 million lobules [114], so that in this setting one leaf node corresponds to about 5^3^ = 125 lobules. The resulting vascular tree is shown in Figure 5.

## 5. Conclusions and Outlook

Even though the coarsest vascular structures in human livers have a large variability, the overall geometric properties are largely invariant between different individuals. Vascular trees generated by the CCO algorithm, that is, constructed to satisfy physical optimality conditions for homogeneous supply, show a certain similarity to measured vascular trees in reality. The similarity can be improved further by a postprocessing step modifying bifurcation angles.

**Limitations**

Several limitations of the methods presented here need to be mentioned.

The main limitation for obtaining more robust radius data is the imaging resolution, but more sophisticated image processing methods can possibly also improve the radius data. Higher resolution image data of small subsamples as described in Section 1 could be used for validation of geometric features at a smaller scale. However, a sufficient number of such datasets were not yet available for this study.

The similarity analysis and corresponding assessment of CCO are here limited to purely geometric features of the vascular structures. One could, for example, consider supplied territories by different parts of the vascular trees. In the model, these could be obtained, for example, by determining watersheds or Voronoï cells. Experimentally one could measure territories where an appropriately injected substance is distributed. However, such measurements in humans in vivo are not feasible. Functional properties as described in Section 2 could also be taken into account for validation of the results. Computing these, however, is not a trivial task and requires well-established and validated additional assumptions. Also, measuring appropriate data is difficult, in particular if in vivo measurements are desired.

We work with a simplified geometric representation of vascular trees allowing only straight cylindrical edges. This is only an approximation of real vascular edges which can be curved and of varying, not necessarily circular, cross-section. Varying out/inflow per leaf node (lobulus) can easily be integrated in the CCO implementation if such data is available. The flow model determining flow resistances does not yet take branching angles into account. The effective influence of the microscopic flow at bifurcations could be used to determine correction factors here.

Finally, the computational performance of our CCO implementation is not satisfactory yet for generating highly detailed vascular trees. This can possibly be remedied by using a multiscale approach partitioning the organ in separate supplied territories.

**Outlook**

Currently, only a single vascular network is generated algorithmically. If PV, HV, and possibly HA are considered simultaneously, penetration of the distinct vascular trees needs to be prevented. This can probably be achieved by considering a joint flow/pressure model also including an effective representation of the organ tissue between the vascular trees following [116] and by adding a nonpenetration constraint in the construction process.

The vascular tree data sets used here are obviously limited to human livers, but the similarity measures and construction algorithm are generic. We plan to apply the methods presented here to in vivo *μ*CT scans of rats and mice and to *μ*CT scans of murine hepatic corrosion casts.

Geometric representation of hepatic vascular trees determined by the methods presented here will be used for flow simulations in the context of multiscale liver modeling.

## Figures and Tables

**Table 1:** Average number of human PV and HV trees considered for statistical analysis that have edges of Strahler* orders 0 to 6, along with the average number of edges per tree where such edges are present.

	Strahler* order	0	1	2	3	4	5	6
PV	No. of trees	167	166	166	164	149	62	5
	avg. no. of edges	5.365	16.771	31.627	67.329	134.134	277.323	408.200
HV	No. of trees	165	163	161	157	152	76	3
	avg. no. of edges	5.242	17.840	39.745	78.720	152.474	272.816	480.667
corr. PV	No. of trees	7	7	7	7	7	7	5
	avg. no. of edges	6.286	39.000	70.429	158.714	369.571	719.143	1183.8

**Table:** (a) Clinical PV

*↗*	0	1	2	3	4	5	6
0	2.0778	1.3015	0.4116	0.4916	0.6231	0.1017	0.0032
1		4.8997	3.8477	1.6626	1.5727	0.4004	0.0193
2			7.7653	10.9897	3.3854	0.6742	0.0181
3				10.3169	25.6297	1.8365	0.0552
4					4.5875	14.5209	0.1550
5						0.3786	1.2481

**Table:** (b) Clinical HV

*↗*	0	1	2	3	4	5	6
0	1.7181	1.8558	0.5686	0.1943	0.4118	0.1045	0.0040
1		4.3881	3.3495	1.1340	1.5930	0.4851	0.0027
2			7.6727	7.9742	3.7217	1.2453	0.0034
3				10.5644	23.4566	2.6166	0.0041
4					5.9190	17.9726	0.0092
5						0.2213	0.7363

**Table:** (c) Corrosion cast PV

*↗*	0	1	2	3	4	5	6
0	0.2833	0.1780	0.0247	0.0509	0.0799	0.0425	0.0084
1		1.7340	0.5611	0.2953	0.4949	0.4139	0.3248
2			2.7724	1.6919	0.8550	0.9524	0.4416
3				5.4717	5.8915	2.2215	1.4351
4					10.0147	20.7643	3.8929
5						9.4238	29.6258

**Table 3:** For different geometric features, the table lists the one-population similarity within the human clinical PV, HV, and corrosion cast PV data sets. Moreover, the two-population similarity between the clinical and corrosion cast PV as well as the clinical PV and HV data sets is listed. Unlike for the individual features, the numerical values of the averages in the one- and two-population cases cannot be compared due to the different averaging formulas (7) and (8).

Feature *f*	Similarity			Similarity	
	PV	HV	corr. PV	PV/corr. PV	PV/HV
Radius *r*	0.270	0.259	0.047	0.174	0.240
Radius decrease *η*_*r*_	0.689	0.706	0.308	0.506	0.670
Radius asymmetry *σ*_*r*_	0.772	0.720	0.238	0.449	0.737
Bif. exponent *γ*	0.895	0.896	0.395	0.684	0.890
Lengths *l*	0.780	0.800	0.386	0.384	0.775
Length decrease *η*_*l*_	0.969	0.968	0.835	0.965	0.969
Length asymmetry *σ*_*l*_	0.925	0.957	0.707	0.937	0.927
Angle *φ*_*a*_	0.923	0.834	0.519	0.810	0.633
Angle *φ*_*b*_	0.938	0.902	0.692	0.908	0.894
Angle *φ*_*c*_	0.872	0.918	0.677	0.827	0.856

Radius average	0.657	0.645	0.247	0.515	0.721
Length average	0.891	0.909	0.643	0.716	0.898
Angle average	0.911	0.885	0.629	0.849	0.794

Total average	0.820	0.813	0.506	0.737	0.812

**Table:** (a) Detailed similarity analysis for clinical PV

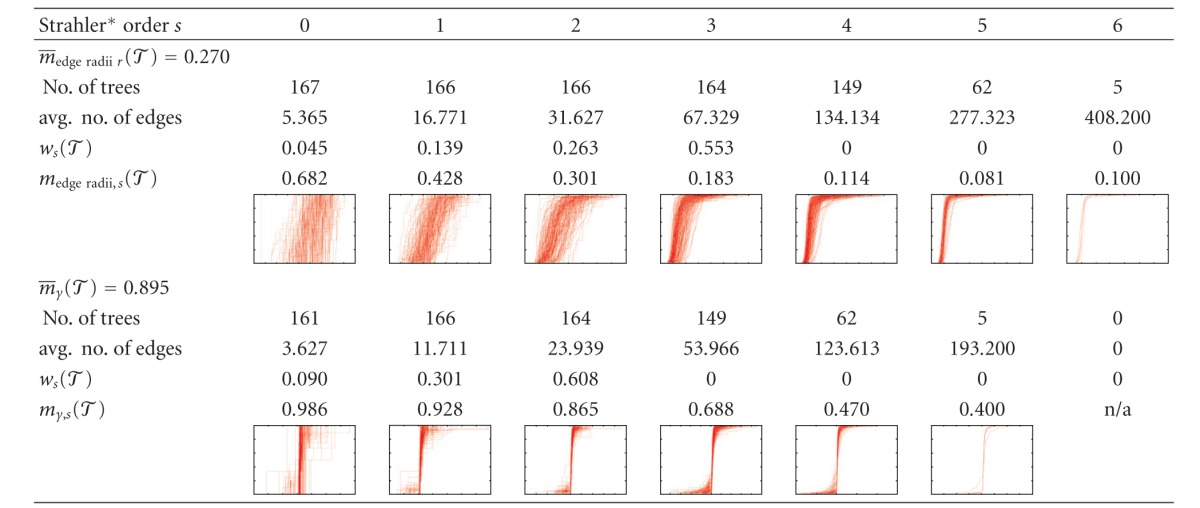

**Table:** (b) Detailed similarity analysis for clinical PV/corrosion cast PV

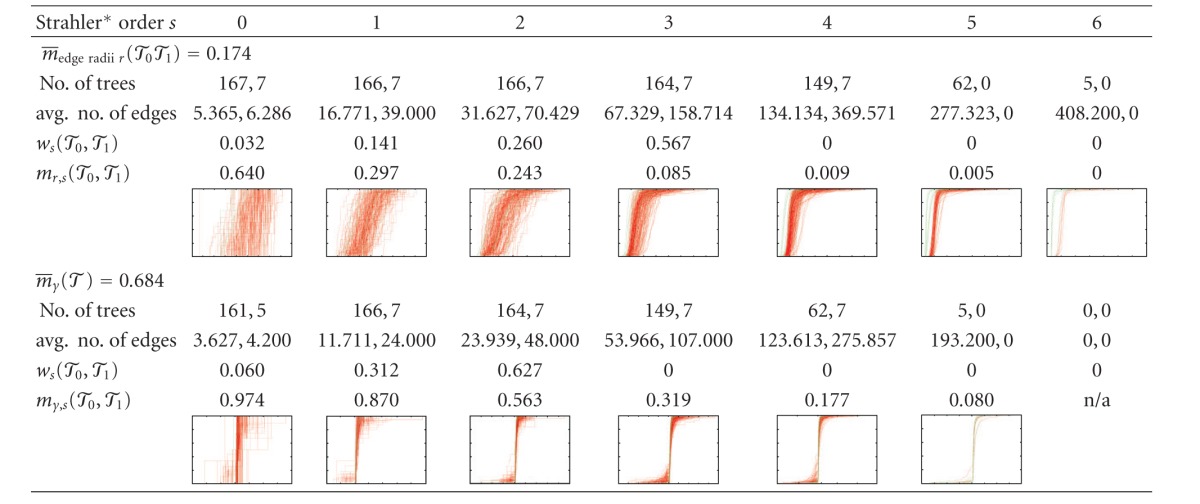

**Table 5:** The best overall CCO results before postprocessing are achieved for a radius exponent *λ* = 2.0 in the cost function (12).

Cost exponent *λ*	1.4	1.6	1.8	2.0	2.2	2.4	2.6
Radius average	0.442	0.428	0.393	0.322	0.333	0.407	0.332
Length average	0.348	0.387	0.460	0.860	0.489	0.405	0.342
Angle average	0.852	0.820	0.748	0.554	0.429	0.498	0.469
Total average	0.560	0.559	0.549	0.603	0.425	0.440	0.386

**Table 6:** In this table we compare the similarity of vascular trees obtained by standard CCO (sCCO) and improved CCO (iCCO) to the clinical CT data sets. The similarity values for individual features, but not the averages, can be compared to the HV and PV single-population similarities in Table 3.

Feature	PV/sCCO	HV/sCCO	PV/iCCO	HV/iCCO
Radius *r*	0.182	0.133	0.182	0.133
Radius decrease *η*_*r*_	0.363	0.246	0.363	0.246
Radius asymmetry *σ*_*r*_	0.376	0.206	0.376	0.206
Bif. exponent *γ*	0.219	0.150	0.219	0.150
Lengths *l*	0.662	0.615	0.614	0.574
Length decrease *η*_*l*_	0.943	0.939	0.902	0.887
Length asymmetry *σ*_*l*_	0.896	0.922	0.849	0.865
Angle *φ*_*a*_	0.444	0.479	0.779	0.581
Angle *φ*_*b*_	0.345	0.212	0.918	0.903
Angle *φ*_*c*_	0.865	0.784	0.875	0.902

Radius average	0.299	0.190	0.299	0.190
Length average	0.845	0.838	0.800	0.787
Angle average	0.545	0.494	0.857	0.802

Total average	0.588	0.542	0.687	0.634
